# Recurrent postpartum chest pain unmasking undiagnosed borderline personality disorder: a diagnostic case report

**DOI:** 10.1186/s12888-025-07307-z

**Published:** 2025-08-28

**Authors:** Aditya Shrivastav, Suhas Tiwaskar, Priyansh Kumar Sahu, Anurag Luharia, Vibhawari Pal, Sarthak Das

**Affiliations:** 1 School of Allied Health Sciences, Datta Meghe Institute of Higher Education and Research, Sawangi, Wardha, Maharashtra 442001 India; 2 Department of Radiodiagnosis, School of Allied Health Sciences, Datta Meghe Institute of Higher Education and Research, Sawangi, Wardha, Maharashtra 442001 India

**Keywords:** Borderline personality disorder, Chest pain, Emergency medicine, Postpartum period, Somatic symptom presentation, Psychosomatic symptoms, Case report

## Abstract

**Background:**

Recurrent Emergency Department (ED) visits for non-cardiac chest pain are common among postpartum women. When cardiac workups are repeatedly normal, psychiatric etiologies, particularly trauma-related personality disorders, should be considered. Borderline Personality Disorder (BPD) may present with panic-like somatic symptoms during periods of interpersonal stress but is often underrecognized in acute care settings.

**Case-presentation:**

We report the case of a 29-year-old postpartum woman who presented to the ED five times over two months with episodic chest pain, palpitations, and a fear of dying. Cardiac and medical evaluations were consistently unremarkable. Each episode followed emotionally distressing interactions with her estranged spouse. The clinical pattern did not meet DSM-5 criteria for primary panic disorder. A structured psychiatric evaluation, corroborated by collateral history, resulted in a diagnosis of BPD based on five core DSM-5 criteria: abandonment fears, affective instability, identity disturbance, impulsivity, and unstable relationships. A sixth criterion, past self-injury, was present but excluded from the diagnostic threshold due to its isolated nature. Treatment included restarting sertraline, continuing propranolol for somatic symptoms, and referral for Dialectical Behaviour Therapy (DBT). The patient reported improved emotional regulation and no further ED visits at one-month followup. While longer-term outcomes remain unknown, her early response was promising.

**Conclusions:**

This case illustrates a diagnostically complex postpartum presentation in which panic-like somatic symptoms masked an underlying personality disorder. Although postpartum depression was initially suspected, psychiatric evaluation revealed previously undiagnosed BPD. Clinicians should consider trauma-informed psychiatric assessment in postpartum patients with medically unexplained symptoms, especially when symptoms are closely tied to interpersonal distress.

**Supplementary Information:**

The online version contains supplementary material available at 10.1186/s12888-025-07307-z.

## Background

Borderline Personality Disorder (BPD) is characterised by pervasive instability in affect regulation, identity, and interpersonal relationships. According to DSM-5, core criteria include frantic efforts to avoid abandonment, affective lability, impulsivity, and recurrent relational conflict [1]. The DSM-5 Alternative Model for Personality Disorders (AMPD) further conceptualises BPD as reflecting impairments in self-functioning (identity, self-direction) and interpersonal functioning (intimacy, empathy) [2]. These domains are often under strain during the postpartum period, marked by biological and psychosocial transition. The perinatal period is a known window of increased psychiatric vulnerability. Women with pre-existing or latent psychiatric conditions may present to Emergency Departments (EDs) with medically unexplained somatic symptoms such as chest pain, palpitations, or dyspnea [3]. While most postpartum ED visits centre on physical complications [4], emerging evidence suggests that somatic complaints can represent masked psychiatric distress, particularly among patients with unrecognised personality pathology [5, 6].

In individuals with BPD, panic-like somatic crises are often situational, emerging in response to perceived rejection or abandonment, and are frequently misdiagnosed as primary panic disorder [5, 7, 8]. Unlike panic disorder, which involves recurrent, unexpected panic attacks and anticipatory anxiety, BPD-related episodes are typically emotionally reactive and context-dependent [1, 8]. While speculative, some studies suggest that hormonal shifts (e.g., estrogen withdrawal), hypothalamic–pituitary–adrenal (HPA) axis hyperreactivity, and circadian dysregulation may contribute to emotional dysregulation in postpartum women predisposed to mood or personality disorders [3]. Although these mechanisms are compelling, they remain speculative and were not directly assessed in this case. Recent literature highlights the diagnostic complexity of perinatal BPD, especially when presenting via somatic symptoms in emergency care [9]. A targeted literature search yielded no previously published case reports linking recurrent postpartum chest pain with a primary diagnosis of BPD in the absence of comorbid mood or anxiety disorders. This diagnostic blind spot underscores the need for trauma-informed psychiatric evaluation in postpartum women presenting with recurrent, medically unexplained symptoms.

## Case presentation

A 29-year-old postpartum woman, working as a primary school teacher, presented to the Emergency Department (ED) five times over two months with recurrent episodes of acute chest tightness, palpitations, and an overwhelming fear of dying. Each episode lasted 15 to 30 min, occurred in the evening, and resolved spontaneously without pharmacological intervention. Notably, each episode followed emotionally charged events, typically after multiple unanswered messages to her estranged spouse, which she interpreted as rejection. The clustering of episodes in the late hours of the day raised the possibility of circadian vulnerability or cumulative diurnal stress.

The onset of symptoms occurred approximately two weeks after the abrupt discontinuation of sertraline 25 mg, which had been initiated during pregnancy for mood symptoms. She reported feeling “emotionally recovered” at the time and discontinued the medication without medical consultation. This discontinuation occurred three months postpartum, in the context of a cesarean delivery and a highly distressing marital separation. She described profound emotional disintegration and identity confusion, stating she often felt “invisible” and no longer knew “who she was.” While the temporal proximity of symptom onset to SSRI discontinuation suggested possible withdrawal syndrome, she did not report classical withdrawal symptoms such as dizziness, nausea, or gastrointestinal upset. Instead, the episodes were consistently triggered by interpersonal conflict and were accompanied by emotional lability and identity disturbance features more characteristic of an underlying Borderline Personality Disorder (BPD) than pharmacologic withdrawal alone.

Her psychiatric history revealed longstanding borderline traits, including impulsive spending, risky sexual behaviour during early adulthood, recurrent emotional outbursts, and intense but unstable romantic relationships. She reported a single episode of superficial self-injury at age 22 but denied further incidents. As this event was isolated, it was considered only a partial fulfilment of the DSM-5 criterion for recurrent suicidal or self-injurious behaviour. She had never undergone a formal psychiatric evaluation but had intermittently used propranolol 10 mg for somatic distress. The sertraline had been discontinued without clinical supervision. Adjustment disorder was briefly considered; however, the chronicity, identity-level disturbance, and pervasive interpersonal dysfunction were inconsistent with a time-limited stress reaction and more reflective of an underlying personality disorder.

**Fig. 1:**
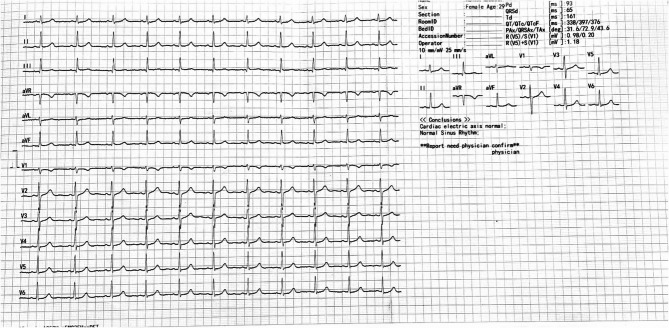
Twelve-lead electrocardiogram obtained during the first emergency department visit showing normal sinus rhythm at a ventricular rate of 81 bpm, normal cardiac axis, and no ST-segment or T-wave abnormalities; no electrocardiographic evidence of myocardial ischemia or acute coronary syndrome

**Fig. 2:**
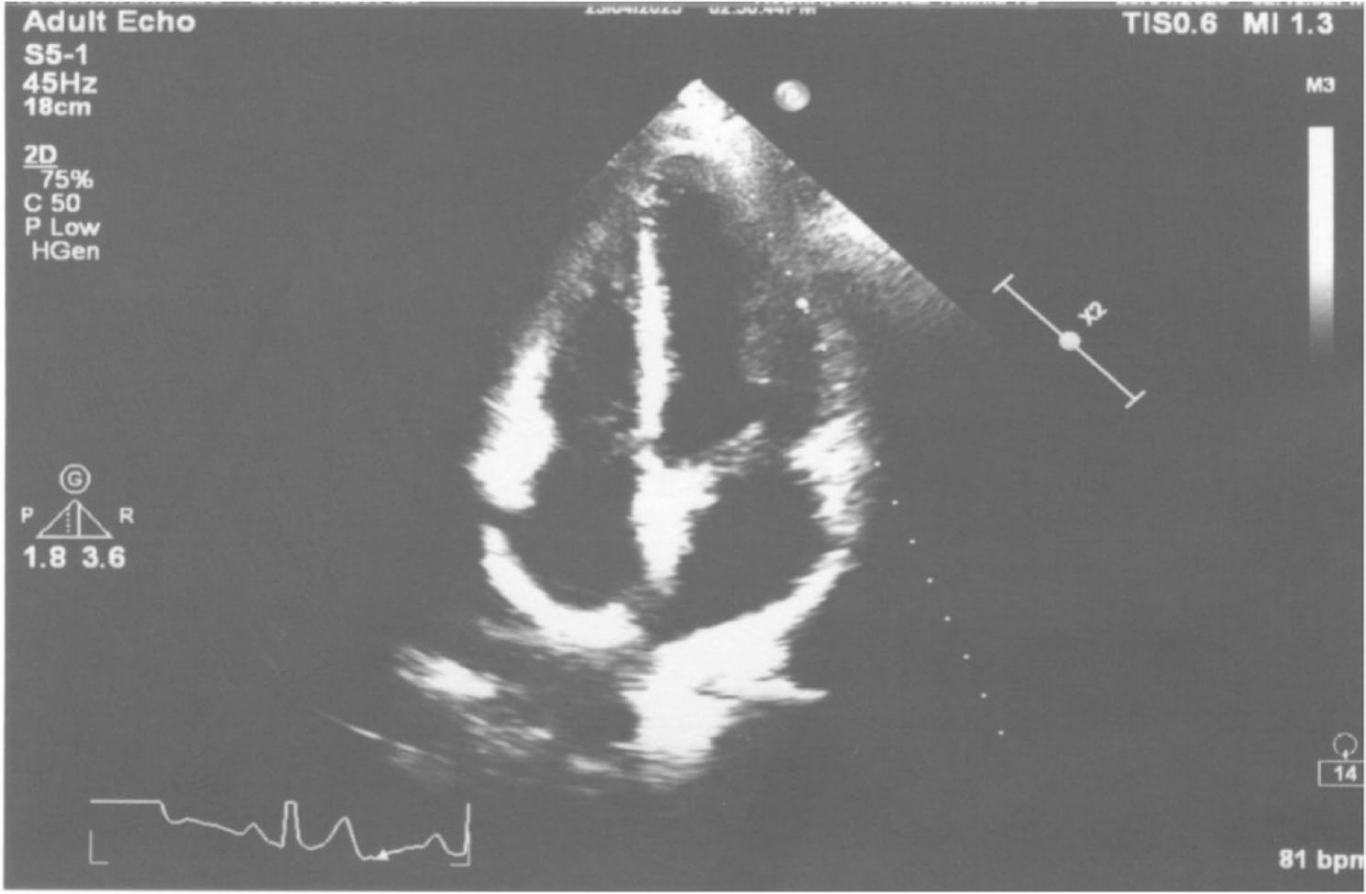
Apical four-chamber transthoracic echocardiogram showing normal chamber morphology and preserved left ventricular systolic function (EF > 55%); no regional wall motion abnormalities, pericardial effusion, or valvular pathology are noted

**Fig. 3:**
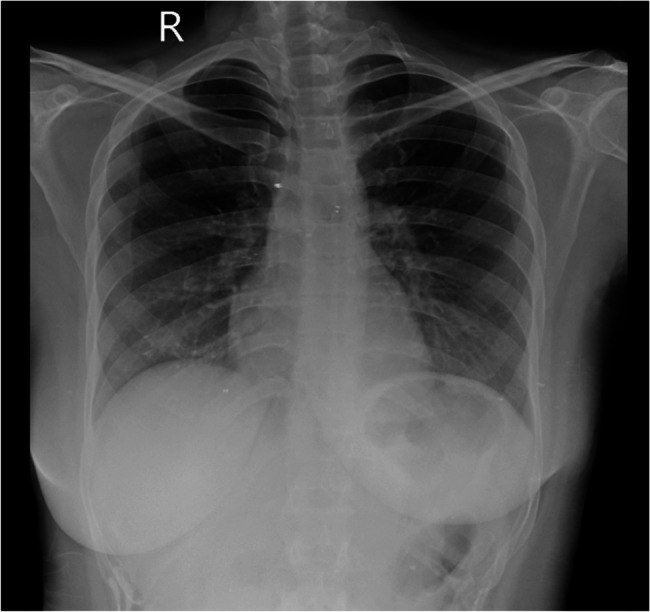
Posteroanterior chest radiograph from the fourth ED visit demonstrating clear lung fields, normal cardiac silhouette, and no evidence of pleural effusion, infiltrate, or pneumothorax

**Fig. 4:**
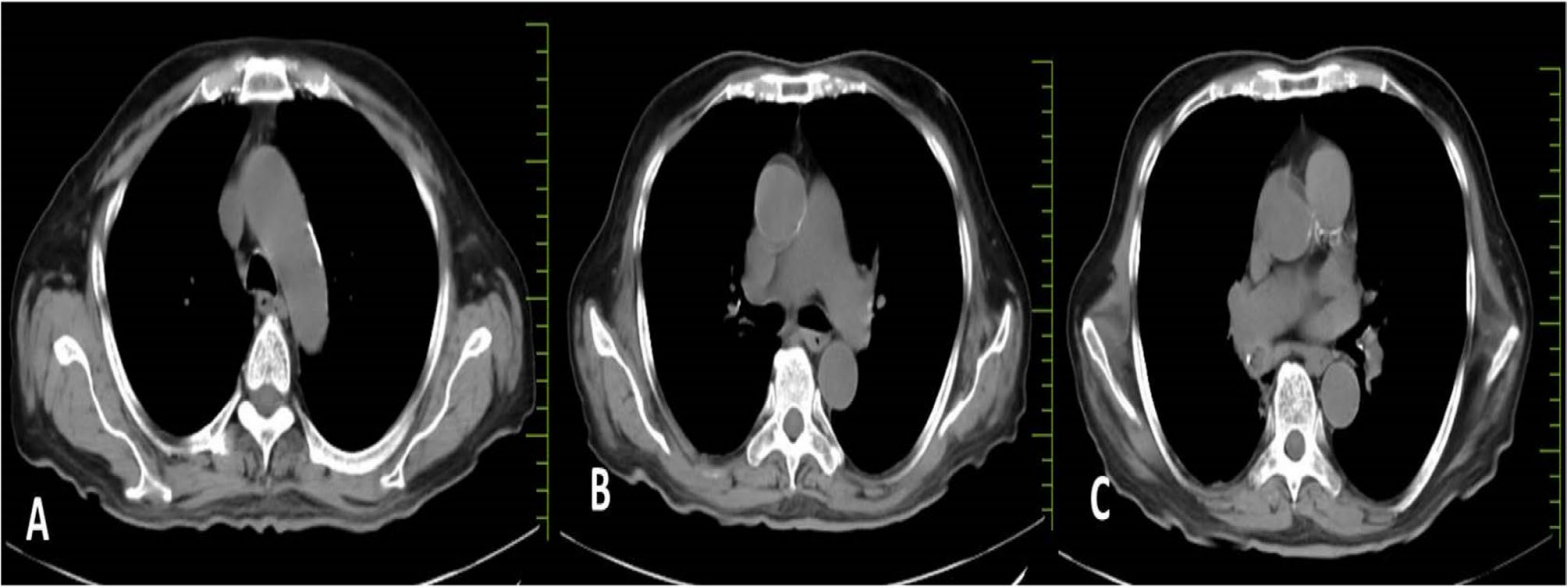
Sequential thoracic CT slices at the level of (**A**) the aortic arch, (**B**) the pulmonary artery bifurcation, and (**C**) the cardiac base. No abnormalities were observed, including no evidence of pulmonary embolism, aortic dissection, pleuroparenchymal disease, or mediastinal mass. These findings contributed to the exclusion of acute cardiopulmonary pathology in the evaluation of recurrent chest pain

Across all five emergency department (ED) visits, the patient’s vital signs remained largely stable, except for mild sinus tachycardia (heart rate 98–120 bpm). Her body mass index (21.4 kg/m²) indicated a healthy weight, making obesity-related cardiovascular risk less likely. Comprehensive evaluations—including complete blood count, metabolic panel, thyroid-stimulating hormone, urine drug screen, troponin-I, brain natriuretic peptide, and D-dimer—were consistently within normal limits. Electrocardiograms demonstrated either sinus rhythm or mild tachycardia without ischemic changes (Fig. 1), and transthoracic echocardiography revealed structurally normal cardiac anatomy with preserved ejection fraction (Fig. 2). Chest radiographs obtained during the first and fourth visits showed no infiltrates, effusions, or cardiomegaly (Fig. 3), while a contrast-enhanced thoracic CT during the fifth visit ruled out pulmonary embolism, aortic dissection, and pleuroparenchymal disease (Fig. 4). Overall, despite recurrent presentations with chest symptoms, all clinical, laboratory, and imaging findings remained unremarkable. Table 1 summarizes the sequence of ED visits, presenting complaints, investigations, and outcomes.

**Table 1:** Chronology of emergency department (ED) visits and workup findings

Visit	Time	Vitals (Heart Rate [bpm]/Blood Pressure [mmHg])	Presenting Symptoms	ECG Findings	2D Echo Findings	Lab/Imaging Workup	Outcome
1st	7:15 PM	104/130/85	Chest tightness, palpitations, overwhelming fear of dying	Sinus tachycardia	Normal	CBC, CMP, Troponin-I, D-dimer, chest X-ray — all within normal limits	Sent home with reassurance
2nd	9:00 PM	110/135/90	Palpitations, dizziness, emotional distress after argument with spouse	Normal sinus rhythm	Normal	ECG, 2D Echo, repeat Troponin-I — normal	Advised psychiatric consult
3rd	6:30 PM	98/125/80	Breathlessness, chest pressure, finger paresthesia, emotional tension	Normal	Normal	TSH, urine drug screen — within normal range	Reassurance given
4th	10:00 PM	120/140/95	Panic, diaphoresis, fear of dying after failed contact with spouse	Sinus tachycardia	Normal	Repeat CBC, chest X-ray, D-dimer — all within normal range	Beta-blocker suggested (propranolol)
5th	8:45 PM	102/128/84	Palpitations, crying spells, chest pressure, derealisation/depersonalisation	Normal	Normal	Contrast-enhanced CT thorax — no abnormalities	Psychiatry referral accepted

During the fifth ED presentation, again triggered by interpersonal conflict involving her child and estranged partner, a psychiatric aetiology was strongly suspected. A consultation–liaison psychiatrist conducted a structured clinical interview, supplemented by collateral input from family members. Based on the DSM-5 diagnostic criteria, the patient met five of the nine required features for Borderline Personality Disorder:

- Frantic efforts to avoid abandonment: Evidenced by escalating communication attempts and repeated ED visits following perceived rejection.
- Affective instability: Marked by rapid emotional shifts in response to interpersonal stress.
- Identity disturbance: She described profound confusion about her identity and persistent feelings of emptiness.
- Impulsivity: Demonstrated through a history of risky sexual behaviour and excessive spending during early adulthood.
- Unstable interpersonal relationships: Characterised by cycles of idealisation and devaluation in romantic attachments.

A sixth criterion, self-injurious behaviour, was partially fulfilled due to a single, non-recurrent episode of superficial self-injury at age 22, and was not counted toward the diagnostic threshold. She did not meet criteria for inappropriate anger or sustained dissociation, though she experienced a brief derealisation episode during the fifth visit, judged to be transient and subclinical.

In addition to meeting categorical DSM-5 criteria, she also fulfilled key elements of the Alternative Model for Personality Disorders (AMPD), including impairments in self-functioning (unstable identity, inconsistent goal-directedness) and interpersonal functioning (heightened rejection sensitivity, difficulty sustaining intimacy). Prominent maladaptive traits included negative affectivity (emotional lability, separation insecurity) and disinhibition (impulsivity). These findings reframed her panic-like somatic episodes as manifestations of affective dysregulation in the context of BPD, rather than a separate panic disorder. However, subclinical panic symptoms may have coexisted.

Management included re-initiation of sertraline, titrated from 25 mg to 50 mg, and continuation of propranolol 10 mg as needed. Clonazepam was intentionally avoided due to the risk of dependence and impulsivity in BPD. Psychoeducation was provided to the patient and her family, emphasising the link between affective dysregulation and somatic symptoms. She was referred for Dialectical Behaviour Therapy (DBT), with a focus on distress tolerance and interpersonal effectiveness.

At follow-ups conducted two and four weeks post-intervention, the patient reported no further ED visits and noted a significant reduction in the frequency and intensity of somatic episodes. She described improved emotional regulation and developing insight into the relational triggers of her distress. These early improvements were attributed to pharmacological stabilisation, increased familial support, and initial therapeutic engagement.

### Patient perspective

“I kept thinking I was having a heart attack, but it was really about feeling abandoned. I did not know emotions could do this to your body.” Long-term outcomes remain unknown and are discussed in the limitations section.

## Discussion

Borderline Personality Disorder (BPD) is under-recognised in emergency care despite its high prevalence among patients presenting with psychiatric symptoms that lack a clear medical aetiology [5, 6, 10]. In this case, repeated Emergency Department (ED) visits for non-cardiac chest pain initially raised suspicion of panic disorder, for which acute management strategies in emergency settings are well described in the literature [11]. However, the episodes were consistently triggered by interpersonal conflict, lacked unpredictability, and were not accompanied by anticipatory anxiety, making a primary diagnosis of panic disorder less likely [1, 8]. While this patient did not meet full criteria for panic disorder, comorbidity between BPD and panic symptoms is well documented, and subclinical panic phenomena may coexist with BPD-related affective dysregulation.

The differential diagnosis included panic disorder (PD), postpartum depression (PPD), generalised anxiety disorder (GAD), somatic symptom disorder (SSD), adjustment disorder, and BPD. PPD was ruled out due to the absence of persistent low mood or anhedonia outside emotionally charged events [12]. GAD was considered unlikely given the lack of pervasive worry or free-floating tension. SSD was also evaluated, but the patient’s interpretation of symptoms lacked illness-related anxiety or preoccupation. Adjustment disorder was briefly considered but deemed insufficient to account for the chronicity, identity-level disturbance, and entrenched relational patterns that emerged well beyond an expected acute stress reaction. Instead, her somatic complaints were consistently temporally linked to perceived interpersonal rejection, aligning more closely with the affective dysregulation seen in BPD [13].

Although the patient reported a single episode of self-injury in early adulthood, it did not meet the threshold for recurrent self-mutilation. Even excluding this partially met criterion, five DSM-5 criteria were fully satisfied: fear of abandonment, affective instability, identity disturbance, impulsivity, and unstable relationships [1]. She did not meet criteria for inappropriate anger or sustained dissociation, although a brief derealisation episode occurred during her fifth ED visit, judged to be transient and subclinical.

In addition to fulfilling the categorical DSM-5 criteria, she also met dimensional criteria under the DSM-5 Alternative Model for Personality Disorders (AMPD), including impairments in identity formation, goal-directedness, rejection sensitivity, and relational instability [2]. Her dominant maladaptive traits included emotional lability, separation insecurity, and impulsivity. These features contextualised her somatic symptoms as affectively reactive responses rather than manifestations of a discrete anxiety disorder.

Comorbidity between BPD and internalising disorders such as depression and anxiety is common, particularly during the perinatal period, where overlapping symptoms may complicate diagnostic clarity [14]. In this case, psychiatric evaluation was only initiated after exhaustive cardiovascular investigations, including electrocardiography, troponin assays, echocardiography, and contrast-enhanced thoracic CT, all of which returned normal findings. Her body mass index was within the normal range, and no cardiovascular risk factors or red flag features were identified. Importantly, although the onset of symptoms followed abrupt SSRI discontinuation, she did not exhibit classical withdrawal features (e.g., dizziness, nausea, gastrointestinal upset), reinforcing the interpretation that the episodes reflected affective dysregulation rather than pharmacologic withdrawal.

Prior studies suggest that up to one-third of patients presenting to EDs with unexplained chest pain have an underlying psychiatric disorder, most often involving anxiety or personality pathology [5, 10]. May et al. (2023) emphasise that personality disorders, particularly in the perinatal period, are frequently overlooked when somatic complaints dominate the clinical picture [9]. To our knowledge, there are no previously published case reports explicitly linking recurrent postpartum chest pain to a primary diagnosis of BPD in the absence of comorbid mood or anxiety disorders. While panic-like episodes are a known feature of BPD, their emergence in the postpartum period, particularly when driving repeated emergency presentations, remains underreported. This case adds novel insight into the complex intersection of relational trauma, somatic expression, and personality dysfunction during a hormonally and psychosocially vulnerable time.

Interestingly, all ED visits occurred in the evening. Though anecdotal, this temporal clustering suggests possible circadian vulnerability or cumulative emotional fatigue across the day. Emerging evidence indicates that diurnal stress rhythms and postpartum hormonal fluctuations may exacerbate emotional dysregulation in at-risk individuals [7].

Clinical improvement followed the re-initiation of sertraline, continuation of propranolol for autonomic symptoms, and referral for Dialectical Behaviour Therapy (DBT). DBT is supported by multiple randomised trials as an effective intervention for emotional dysregulation, suicidality, and impulsivity in individuals with BPD [15]. Psychoeducation and increased family engagement may also have contributed to the reduction in ED utilisation. However, the short follow-up duration limits conclusions regarding the long-term trajectory. Structured screening tools such as the McLean Screening Instrument for BPD (MSI-BPD) or the Personality Diagnostic Questionnaire-4+ (PDQ-4+) were not employed due to time constraints in the emergency setting. Nevertheless, future use of such tools may facilitate early recognition of personality pathology in postpartum patients presenting with recurrent, unexplained physical symptoms [14, 16].

As Paris notes, effective treatment of BPD requires more than symptom control; it necessitates sustained, trauma-informed engagement that addresses the underlying relational vulnerabilities, identity instability, and emotional dysregulation at the core of the disorder [17]. This case highlights the importance of considering underlying personality pathology in postpartum women presenting with recurrent somatic complaints, particularly when symptoms are emotionally reactive and closely tied to interpersonal distress. A trauma-informed, dimensional diagnostic framework may help reduce diagnostic delays, minimise unnecessary medical interventions, and guide timely, person-centred psychiatric care.

## Limitations

This case report has several limitations. First, the diagnosis of Borderline Personality Disorder (BPD) was made without the use of standardised screening instruments such as the McLean screening Instrument for BPD (MSI-BPD) or the Personality Diagnostic Questionnaire-4+ (PDQ-4+), which are commonly used to support structured personality assessment. Their omission reflects the pragmatic constraints of emergency psychiatry, where evaluations prioritise immediate stabilisation and safety. Nevertheless, the lack of validated tools limits objective confirmation, especially in personality disorders where symptom overlap and diagnostic ambiguity are common. Future case series should consider incorporating such instruments during outpatient followup, particularly in high-risk postpartum populations presenting with recurrent somatic complaints.

Second, while collateral history was obtained and the clinical features met DSM-5 criteria for BPD, the psychiatric formulation was based primarily on a single clinical interview. This limits diagnostic depth and reliability. In BPD, emotional reactivity and unstable self-narratives can compromise diagnostic clarity, and the patient’s prepartum psychiatric trajectory was incompletely documented. This leaves gaps in understanding personality traits’ chronicity and developmental pattern, which is essential for robust diagnosis. Similarly, although postpartum depression was clinically ruled out based on symptom patterns, no standardised mood screening instrument such as the Edinburgh Postnatal Depression Scale (EPDS) was administered. This may reduce diagnostic precision and should be addressed in future clinical protocols.

Third, the potential contribution of SSRI discontinuation syndrome could not be fully excluded. While the temporal proximity of abrupt sertraline cessation raised this possibility, the patient’s symptoms were consistently triggered by interpersonal conflict. They were more characteristic of BPD-related affective dysregulation than pharmacological withdrawal. Nonetheless, the absence of neurovegetative or sensory withdrawal symptoms does not entirely preclude a contributory role. Additionally, the duration of clinical followup limited to two and four weeks is insufficient to assess sustained emotional stability, treatment adherence, or relapse risk. Although the patient showed early improvement with pharmacological re-initiation and referral to Dialectical Behaviour Therapy (DBT), longer-term outcomes such as therapeutic engagement, emotional resilience, and healthcare utilisation remain unknown.

Fourth, no hormonal or neuroendocrine markers (e.g., estradiol, cortisol) were measured, limiting the ability to explore biological contributors to postpartum affective lability. While hypothalamic–pituitary–adrenal (HPA) axis reactivity and estrogen withdrawal have been implicated in BPD vulnerability, these mechanisms remain speculative and were not directly evaluated.

Finally, as a single-case report, these findings are not generalisable to the wider postpartum population. Nonetheless, the case offers clinically relevant insight into a diagnostically complex presentation, highlighting the need for trauma-informed psychiatric assessment when medically unexplained somatic symptoms occur in emotionally vulnerable postpartum patients.

## Conclusion

This case underscores the diagnostic complexity of panic-like somatic symptoms in the postpartum period, particularly when rooted in unrecognised Borderline Personality Disorder (BPD). Despite multiple emergency department visits and normal cardiac evaluations, the patient’s symptoms were clinically consistent with affective dysregulation and interpersonal distress characteristic of BPD. While abrupt SSRI discontinuation and postpartum mood vulnerability may have contributed, the longitudinal pattern and interpersonal context supported a primary personality-based formulation. Early improvement following psychiatric intervention highlights the value of timely recognition, trauma-informed assessment, and integrated care planning. Clinicians should maintain a high index of suspicion for personality pathology in postpartum women presenting with recurrent, medically unexplained physical complaints, especially when symptoms are temporally linked to perceived rejection or emotional conflict. Incorporating routine screening and structured psychiatric evaluation in high-risk populations may help reduce diagnostic delay, avoid unnecessary investigations, and improve patient outcomes.

## Supplementary Information

Supplementary Material 1.

## Data Availability

The data supporting the findings of this case report are available from the corresponding author upon reasonable request.

## Abbreviations

Abbreviation: Full Form
AMPD: Alternative Model for Personality Disorders
BPD: Borderline Personality Disorder
BMI: Body Mass Index
BP: Blood Pressure
CBC: Complete Blood Count
CMP: Comprehensive Metabolic Panel
CT: Computed Tomography
DBT: Dialectical Behaviour Therapy
DSM-5: Diagnostic and Statistical Manual of Mental Disorders, Fifth Edition
ECG: Electrocardiogram
ED: Emergency Department
EF: Ejection Fraction
EPDS: Edinburgh Postnatal Depression Scale
GAD: Generalized Anxiety Disorder
HPA axis: Hypothalamic–Pituitary–Adrenal Axis
HR: Heart Rate
LV: Left Ventricle
MSI-BPD: McLean Screening Instrument for Borderline Personality Disorder
PD: Panic Disorder
PDQ-4+: Personality Diagnostic Questionnaire–4+
PPD: Postpartum Depression
PRN: Pro Re Nata (as needed)
SSD: Somatic Symptom Disorder
SSRI: Selective Serotonin Reuptake Inhibitor
TSH: Thyroid-Stimulating Hormone
UDS: Urine Drug Screen
