# Supplementary material for: Recurrent postpartum chest pain unmasking undiagnosed borderline personality disorder: a diagnostic case report
Source: BMC Psychiatry. 2025 Aug 28;25:838. doi: 10.1186/s12888-025-07307-z (PMC12395791; doi:10.1186/s12888-025-07307-z)
Supplement: Supplementary file 1 — Supplementary Material 1. [file 12888_2025_7307_MOESM1_ESM.pdf]

CAREChecklistofinformationtoincludewhenwritingacasereport

(cc) BY-NC-ND

Topic	Item	Checklistitemdescription	ReportedonLine
**Title**	1	Thediagnosisorinterventionofprimaryfocusfollowedbythewords“casereport”	Page 1, Line 1
**KeyWords**	2	2to5keywordsthatidentifydiagnosesorinterventionsinthiscasereport,including"case report"	Page 1, Line 22–23
**Abstract (noreferences)**	3a	Introduction:Whatisuniqueaboutthiscaseandwhatdoesitaddtothescientificliterature?	Page 1, Line 6–11
	3b	Mainsymptomsand/orimportantclinicalfindings	Page 1, Line 12–18
	3c	Themaindiagnoses,therapeuticinterventions,andoutcomes	Page 1, Line 18–26
	3d	Conclusion—Whatisthemain“take-away”lesson(s)fromthiscase?	Page 1, Line 26–31
**Introduction**	4	Oneortwoparagraphssummarizingwhythiscaseisunique(**mayinclude**reference**s**)	Page 1, Line 34 – Page 2, Line 22
**PatientInformation**	5a	De-identifiedpatientspecificinformation	Page 2, Line 25–27
	5b	Primaryconcernsandsymptomsofthepatient	Page 2, Line 28–33
	5c	Medical,family,andpsycho-socialhistoryincludingrelevantgeneticinformation	Page 2, Line 34 – Page 3, Line 6
	5d	Relevantpastinterventionswithoutcomes	Page 3, Line 7–11
**Clinical Findings**	6	Describesignificantphysicalexamination(PE)andimportantclinicalfindings	Page 3, Line 12–20
**Timeline**	7	Historicalandcurrentinformationfromthisepisodeofcareorganizedasatimeline	Page 4, Line 1–19 (Table 1)
**Diagnostic Assessment**	8a	Diagnostictesting(suchasPE,laboratorytesting,imaging,surveys)	Page 3, Line 12–20 and Page 4, Table 1
	8b	Diagnosticchallenges(suchasaccess to testing,financial,or cultural)	Page 3, Line 21–22
	8c	Diagnosis (including other diagnoses considered)	Page 3, Line 23–27
	8d	Prognosis(suchasstaginginoncology)whereapplicable	Page 3, Line 37 – Page 4, Line 3
**Therapeutic Intervention**	9a	Typesoftherapeuticintervention(suchaspharmacologic,surgical,preventive,self-care)	Page 3, Line 28–36
	9b	Administrationoftherapeuticintervention(suchasdosage,strength,duration)	Page 3, Line 28–36
	9c	Changesintherapeuticintervention(withrationale)	Page 3, Line 35–36
**Follow-upand Outcomes**	10a	Clinicianandpatient-assessedoutcomes(ifavailable)	Page 4, Line 4–10
	10b	Importantfollow-updiagnosticandothertestresults	Page 4, Line 4–10
	10c	Interventionadherenceandtolerability(Howwasthisassessed?)	Page 4, Line 4–10
	10d	Adverseandunanticipatedevents	Not applicable
**Discussion**	11a	AscientificdiscussionofthestrengthsANDlimitationsassociated with thiscasereport	Page 4, Line 12 – Page 5, Line 33
	11b	Discussionofthe relevantmedicalliterature**withreferences**	Page 4, Line 12 – Page 5, Line 33
	11c	Thescientificrationaleforanyconclusions(includingassessmentofpossiblecauses)	Page 5, Line 10–25
	11d	Theprimary“take-away”lessonsofthiscasereport(withoutreferences)inaoneparagraphconclusion	Page 5, Line 26–33
**PatientPerspective**	12	Thepatientshouldsharetheirperspectiveinonetotwoparagraphsonthetreatment(s)theyreceived	Optional (Not mentioned)

**InformedConsent**	**13**	Didthepatientgiveinformedconsent?Pleaseprovideifrequested......................................	**Yes**
